# Supplementary material for: Defect-driven antiferromagnetic domain walls in CuMnAs films
Source: Nat Commun. 2022 Feb 7;13:724. doi: 10.1038/s41467-022-28311-x (PMC8821625; doi:10.1038/s41467-022-28311-x)
Supplement: Supplementary file 1 — Supplementary Information [file 41467_2022_28311_MOESM1_ESM.pdf]

Supplementary information

for

Defect-driven antiferromagnetic domain walls in CuMnAs films

Sonka Reimers,[*,†,‡] Dominik Kriegner,[¶,§] Olena Gomonay,[‖] Dina Carbone,[⊥] Filip Krizek,[§] Vit Novák,[§] Richard P. Campion,[†] Francesco Maccherozzi,[‡] Alexander Björling,[⊥] Oliver J. Amin,[†] Luke X. Barton,[†] Stuart F. Poole,[†] Khalid A. Omari,[†] Jan Michalička,[#] Ondřej Man,[#] Jairo Sinova,[‖] Tomáš Jungwirth,[§,†] Peter Wadley,[†] Sarnjeet S. Dhesi,[*,‡] and Kevin W. Edmonds[*,†]

†*School of Physics and Astronomy, University of Nottingham, Nottingham NG7 2RD, United Kingdom*

‡*Diamond Light Source, Chilton OX11 0DE, United Kingdom*

¶*Institut für Festkörper- und Materialphysik and Würzburg-Dresden Cluster of Excellence ct.qmat, Technische Universität Dresden, 01062 Dresden, Germany*

§*Institute of Physics, Czech Academy of Sciences, 162 00 Praha 6, Czech Republic*

‖*Institut für Physik, Johannes Gutenberg Universität Mainz, 55099 Mainz, Germany*

⊥*MAX IV Laboratory, Lund University, 22100 Lund, Sweden*

#*Central European Institute of Technology, Brno University of Technology, 612 00, Brno, Czech Republic*

E-mail: Sonka.Reimers@nottingham.ac.uk; Sarnjeet.Dhesi@diamond.ac.uk; Kevin.Edmonds@nottingham.ac.uk

Supplementary Note 1 – Polarised X-ray spectroscopy

The antiferromagnetic (AF) domains and microtwin defects were imaged using PhotoEmission Electron Microscopy (PEEM) with magnetic contrast obtained using X-ray Magnetic Linear Dichroism (XMLD) and defect contrast obtained using X-ray Linear Dichroism (XLD). The characteristic Mn $L_{2,3}$ XMLD spectrum was determined using an exchange-coupled Fe/CuMnAs bilayer in which the AF spin axis could be rotated using a field of 0.1 T[1] via exchange coupling to the Fe thin film. Supplementary Fig. S1 shows X-ray absorption spectroscopy (XAS), XMLD and XLD measurements over the Mn $L_{2,3}$ edge. The spectra were recorded using a spatially averaging total electron yield probe. The XMLD spectra were obtained as the difference between XAS spectra recorded with the x-ray polarization (**E**) parallel and perpendicular to the AF spin axis aligned along either [010] (Supplementary Fig. S1 **b**, blue curve) or [110] (Supplementary Fig. S1 **b**, red curve). Tetragonal CuMnAs films exhibit a pronounced XLD for different orientations of **E** with respect to the [001] axis. The XLD spectrum shown in Supplementary Fig. S1 **c** is the difference between XAS spectra recorded with **E** perpendicular to and at 15° to the [001] axis.

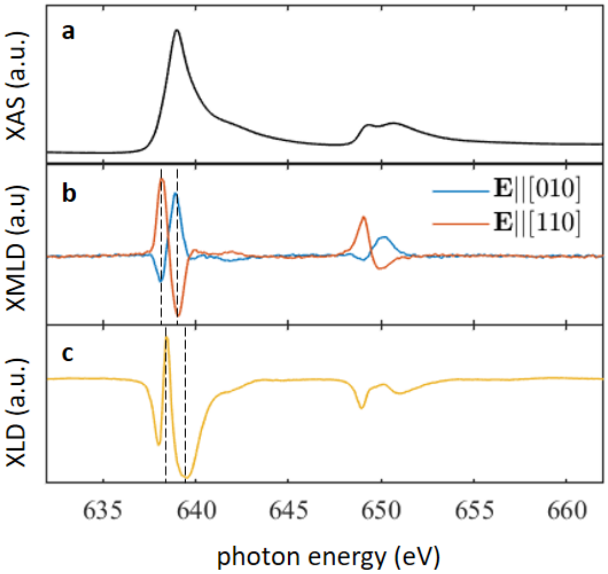

Supplementary Fig. S1: Mn $L_{2,3}$ XAS, XMLD and XLD spectra from CuMnAs. **a**, XAS spectrum. **b**, XMLD for **E** ∥ [010] (blue) and **E** ∥ [110] (red). **c**, non-magnetic XLD spectrum. The vertical dashed lines in b and c indicate the photon energies used for magnetic domain and microtwin defect PEEM imaging, respectively.

Supplementary Note 2 – Imaging of antiferromagnetic domain walls in CuMnAs using X-ray magnetic linear dichroism

In Supplementary Fig. S2, we illustrate how a 90° and 180° domain wall (DW) in a CuMnAs film can be identified using XMLD-PEEM imaging. Supplementary Figs. S2 **a** and **b** show an area of a CuMnAs film imaged with **E** ∥ [110] and **E** ∥ [010], respectively. The area features both 90° DWs, marked with orange broken lines, and 180° DWs, marked with green broken lines. We observe that a 180° DW can split into two 90° DWs.

Supplementary Figs. S2 **c** - **f** show calculated XMLD intensity profiles[1,2] for a 90° DW (panels c and e) and a 180° DW (panels d and f). A 90° DW separates dark and light areas in XMLD-PEEM images with **E** ∥ [110]. In XMLD-PEEM images with **E** ∥ [010] these DWs show up as either dark or light lines depending on the spin axis in the center of the wall (here shown for a light wall with the spin axis in the center of the domain wall along [010]).

A 180° DW appears as a single color line when imaged with **E** ∥ [110]. However, as the Néel vector undergoes a full 180° rotation, for **E** ∥ [010] both maximum and minimum XMLD contrast is achieved locally on the DW. This allows such DWs to be clearly identified in the XMLD-PEEM images.

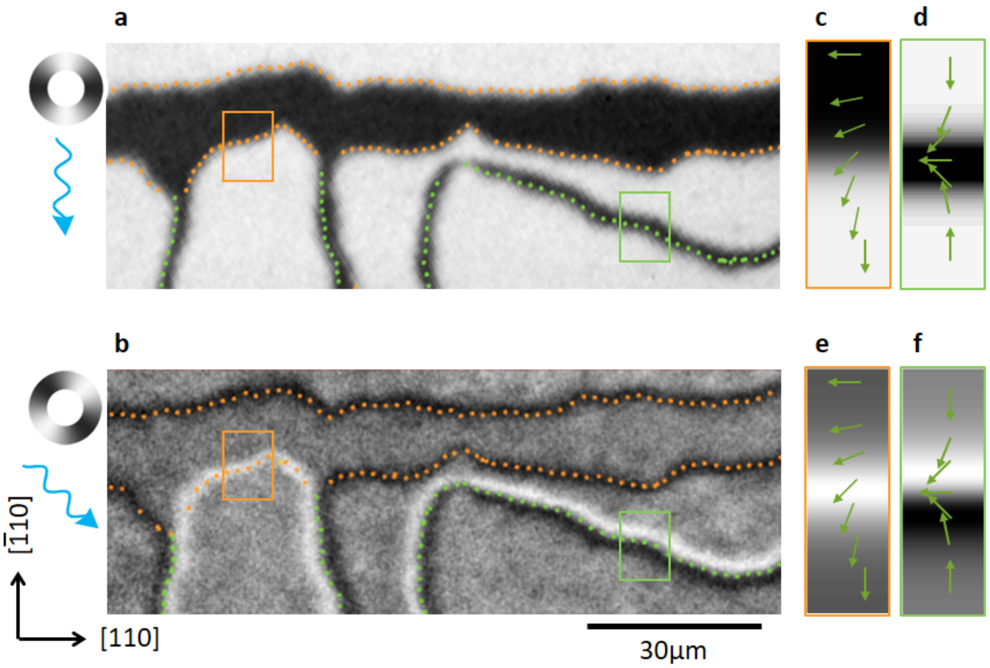

Supplementary Fig. S2: XMLD-PEEM imaging of AF domain walls. **a**, XMLD-PEEM image with **E** || [110]. 90° and 180° domain walls are marked with orange and green broken lines, respectively. **b**, XMLD-PEEM image of the same area as panel a, but with **E** || [010]. The blue arrows indicate the direction of the incident X-ray beam and the greyscale wheels indicate the direction of the local spin axis. **c, d**, Calculated XMLD intensity profiles across a 90° DW (c, orange frame) and a 180° DW (d, green frame) for **E** || [110]. The green arrows illustrate the rotation of the Néel vector across the wall. **e, f**, Same as c and d, but for **E** || [010]. Examples of corresponding intensity profiles in the experimental images are marked with rectangular boxes with matching frame colors in panels a and b.

Supplementary Note 3 – Scanning X-ray diffraction microscopy

The 003 reciprocal space map (RSM) features diffuse scattering along the $q_{\langle 101\rangle}$-type directions and sharper wings along the $q_{\langle 110\rangle}$-type directions in addition to fringes[3] along the $q_{[001]}$ direction arising from the finite film thickness. See Supplementary Movie. The diffuse scattering along the $q_{\langle 101\rangle}$-type directions has been attributed to $c/2$-slip dislocations[4]. The sharper wings result from microtwin defects running along the {111} planes. The wings are not produced by the crystalline structure of the microtwin defects, as they have a different crystal orientation, but arise from changes imposed by the defects on the surrounding (001) oriented lattice. The RSM was obtained from an area of the sample that contained only three out of the four microtwin types. Consequently, the RSM has only three wings.

As shown in Fig. 2 b of the main text, real space intensity maps of a specific wing show a set of defect lines running perpendicular to the direction of the selected wing. A real space map produced from the intensity of all the wings (see Fig. 2 c in the main text) reveals a pattern of defect lines running parallel to [110] and $[1\bar{1}0]$ which matches the microtwin defect pattern determined using atomic force microscopy[4]. The RSM of a particular defect line has a single wing around the diffraction peak, implying a one-to-one correspondence between the orientation of the microtwin and the direction of the wing in reciprocal space, which are attributed to lattice tilts around the microtwin.

Supplementary Fig. S3 shows examples of the diffraction images used to generate the SXDM microtwin maps. Supplementary Figs. S3 **a** - **c** show diffraction images summed over the sample surface for the Bragg condition ($\Theta = \Theta_{\mathrm{Bragg}}$) and with the sample rotated away from the Bragg angle by $\Delta\Theta = \pm 0.4°$.

The geometry for $\Theta = \Theta_{\mathrm{Bragg}} + \Delta\Theta$ is shown in Supplementary Fig. S3**d** illustrating how the isosurface of the CuMnAs 003 RSM can be sliced by the detector plane shown as the grey sheet. The schematic shows that for this particular geometry the detector plane slices part of the fringes along $q_{[001]}$ and two of the microtwin defect wings. The configuration for $\Theta = \Theta_{\mathrm{Bragg}} - \Delta\Theta$ can be obtained via symmetry and slices the other two microtwin wings.

During an 2D-mesh scan with $\Theta = \Theta_{\mathrm{Bragg}} + \Delta\Theta$ we thus observe increased intensity in particular areas of the detector when a microtwin is within the illuminated area and lower intensity in a microtwin-free area, as shown in Supplementary Fig. S3 **e-g**. Mapping the intensity of the relevant detector area against sample position during an 2D-mesh scan for $\Theta = \Theta_{\mathrm{Bragg}} + \Delta\Theta$ thus allows imaging of two types of microtwins. The other two types can be imaged for $\Theta = \Theta_{\mathrm{Bragg}} - \Delta\Theta$.

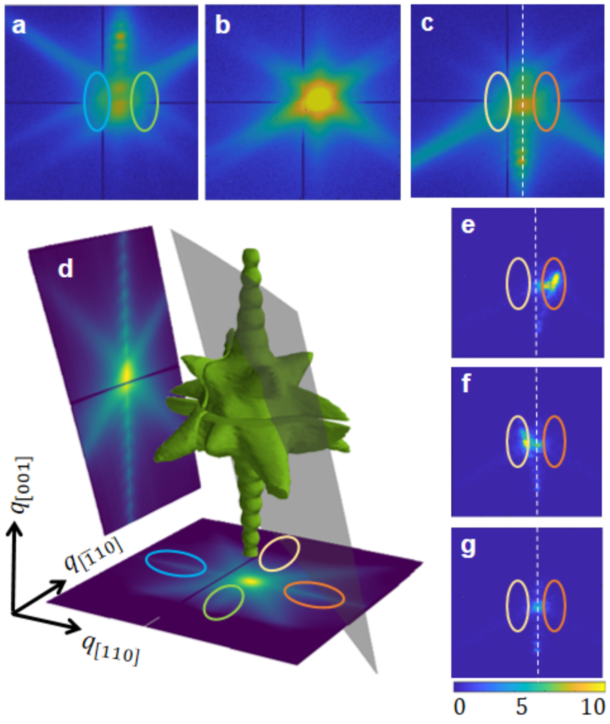

Supplementary Fig. S3: SXDM microtwin mapping. **a-c**, Spatially-averaged diffraction images on a log-scale for three different scattering geometries: **a**, $\Theta = \Theta_{\mathrm{Bragg}} - \Delta\Theta$; **b**, $\Theta = \Theta_{\mathrm{Bragg}}$; **c**, $\Theta = \Theta_{\mathrm{Bragg}} + \Delta\Theta$. **d**, RSM of the 003 Bragg reflection from an area with microtwin defects. The grey sheet indicates the detector plane for $\Theta = \Theta_{\mathrm{Bragg}} + \Delta\Theta$ which slices through two of the wings arising from the microtwin defects. **e-g**, Spatially-resolved diffraction images on a linear scale for $\Theta = \Theta_{\mathrm{Bragg}} + \Delta\Theta$: **e**, on one microtwin; **f**, on a microtwin with a different orientation; **g**, from an area without microtwins. The color bar represents the counts in e-g. The broken white lines serve as guides to the eye. The colored ovals in (d) indicate the areas of reciprocal space used to map the microtwin defects and are also marked by the corresponding colored ovals in the rest of the figure.

Supplementary Note 4 – Micromagnetic simulations

The simulations of the antiferromagnetic domain walls are based on static Landau-Lifshitz-Gilbert equations for two magnetic sublattices. The magnetic energy of the antiferromagnetic film (per unit volume) consists of three principal contributions: the magnetic anisotropy of bulk tetragonal CuMnAs (w_{an}), the magnetic anisotropy of the microtwin (w_{tw}) and the magnetoelastic energy (w_{me}) which includes the defect-induced strain.

Based on the tetragonal symmetry of CuMnAs, the magnetic anisotropy of the bulk film is given by

$$w_{\mathrm{an}} = \frac{1}{2} M_s H_{an}^{\parallel} n_z^2 - \frac{1}{4} M_s H_{an}^{\perp} (n_x^4 + n_y^4), \tag{1}$$

where $\mathbf{n}$ is the reduced Néel vector ($|\mathbf{n}| = 1$), whose direction coincides with the direction of the spin axes, $M_s/2$ is the sublattice magnetization, and the positive constants $H_{an}^{\parallel}$ and $H_{an}^{\perp}$ correspond to the out-of-plane and in-plane magnetic anisotropy fields. The $x(y)$-axis is parallel to the magnetic easy axis along [110] ([1$\bar{1}$0]).

To define w_{tw} and w_{me} we first describe a microtwin as a local, coherent rotation of the bulk CuMnAs. The lattice of the microtwin is a reflection of the bulk lattice in a {111} mirror plane, *i.e.* the twinning plane. The twinning plane makes an angle of $\psi = \arctan(c/\tilde{a})$ with the (001) plane, where $\tilde{a} = \sqrt{2}a$ and $a(c)$ are the lattice parameters along the [100]([001]) directions (see Supplementary Fig. S4 **a**). Substituting $a = 3.853$ Å and $c = 6.278$ Å gives $\psi = 49°$. For tetragonal symmetry, it follows that there are two possible twins for each of the (111) and ($\bar{1}$11) mirror planes. In each case the twin plane makes an angle of $\pm\psi$ or $\pi \pm \psi$ with the film plane, giving four possible microtwin orientations. The lattice vectors within the microtwin region are rotated by $\pi - 2\psi \approx 82°$ with respect to the bulk lattice. (see Supplementary Fig. S4).

The magnetic anisotropy of a microtwin, w_{tw}, is calculated using Eq. (1) after taking into account a lattice rotation of 2ψ so that $x' = x\cos(\pi - 2\psi) + z\sin(\pi - 2\psi)$ and $z' =$

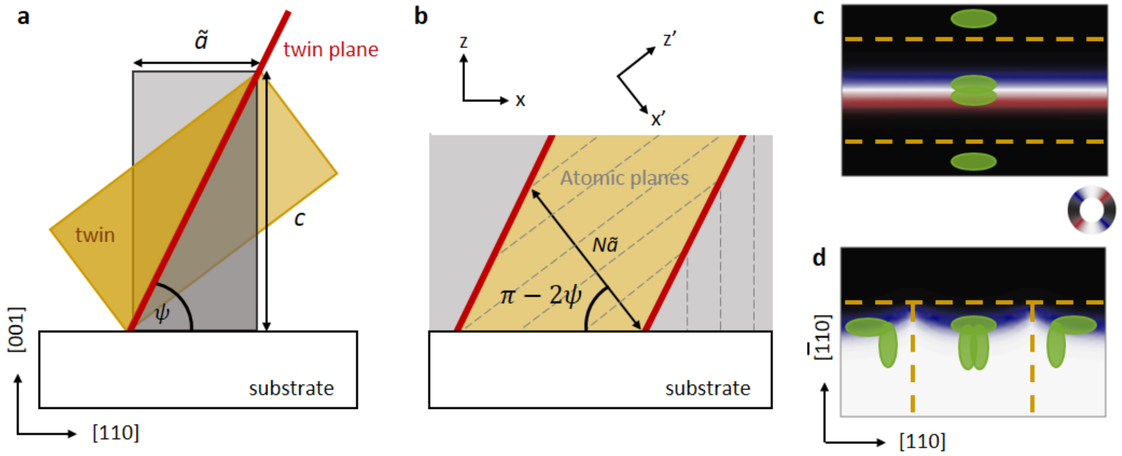

Supplementary Fig. S4: Strain field created by microtwin defects a, Schematic (not to scale) of the CuMnAs unit cell (grey), with the in-plane parameter $\tilde{a}$ (measured along the [110] direction) and out-of plane lattice parameter, c. The rotated unit cell of the microtwin defect is shown as the yellow rectangle. The twinning plane (solid red line) is the {111} mirror plane, forming an angle of ψ with the substrate interface. **b**, Microtwin mismatch at the film-substrate interface. Inside the microtwin, N atomic planes have a width of $N\tilde{a}$ which is smaller than the projection of the microtwin on the film plane, leading to a mismatch at the substrate interface. x,z and x', z' co-ordinates refer to the bulk CuMnAs and microtwin reference frames, respectively.

$-x\sin(\pi-2\psi)+z\cos(\pi-2\psi)$. Since $H_{an}^{\parallel} \gg H_{an}^{\perp}$, we find that

$$w_{\rm tw} = \frac{1}{2}M_s H_{an}^{\parallel}\cos^2(2\psi)n_z^2 + \frac{1}{2}M_s H_{an}^{\parallel}\sin^2(2\psi)n_x^2 - \frac{1}{4}M_s H_{an}^{\perp} n_y^4 + \dots \quad (2)$$

The second term removes the degeneracy between the x and y axes and implies that an orientation of the Néel vector perpendicular to the projection of the microtwin on the film plane is energetically unfavourable. In equation (2) we have omitted the higher order terms.

To calculate the strain induced by a defect and the corresponding contribution to the magnetoelastic energy we consider the mismatch at the interface (between the CuMnAs microtwin and the GaP substrate) that a rotation of the microtwin lattice induces, as shown in Supplementary Fig. S4 **b**. The projection of the microtwin on the film plane, $d_{\rm tw}/\cos(\pi-2\psi)$, is larger than the microtwin width $d_{\rm tw}=N\tilde{a}$, where N is the number of atomic planes. The resulting structural defect can be interpreted as an edge dislocation localized at the

interface between the microtwin boundaries (parallel to the y-axis), with a Burgers' vectors ($\mathbf{b}$) parallel to the x-axis. Such a defect has a constant dislocation density given by

$$\alpha_{xy}(x,y) = \tilde{a}\left(\frac{1}{\cos(\pi - 2\psi)} - 1\right)\Theta(x,y) = \tilde{a}\frac{c^2 - \tilde{a}^2}{c^2 + \tilde{a}^2}\Theta(x,y) \tag{3}$$

where the form-function $\Theta(x,y) = 1$ inside the microtwin and zero elsewhere. Equation 3 is invariant upon the transformation $\psi \to (\pi - \psi)$, *i.e.* defects with opposite tilts are assumed to have the same dislocation density. Assuming further that the microtwin thickness is much smaller than the domain wall width we can consider a microtwin as an edge disclocation with an effective Burgers' vector $b_x = N\alpha_{xy}$.

According to elasticity theory[5] an edge dislocation creates an additional strain field $u^{\mathrm{tw}}_{jk}(x,z)$ whose distribution is calculated based on the standard equations for the displacement vector $\mathbf{u}$ which shows a jump $u_x(z+0) - u_x(z-0) = b_x$ at the interface between the substrate and microtwin region. Far from the dislocation core, $u^{\mathrm{tw}}_{jk}(x,z)$ decays as $\propto |\mathbf{b}|/\rho$ with the distance, ρ, from the defect.

We neglect the weak space dependence of u^{tw}_{jk} for a singular defect and consider only the leading contribution into magnetic anisotropy given by the u^{tw}_{xx} component. We model the strain field with a constant function $u^{\mathrm{tw}}_{xx} = u_0$ for the microtwins aligned along the y-axis and $u^{\mathrm{tw}}_{yy} = u_0$ for the microtwins aligned along the x-axis. The strain field of two parallel y-axis oriented defects is doubled, $u^{\mathrm{tw}}_{xx} = 2u_0$, between the defects and $u^{\mathrm{tw}}_{xx} = u_0$ outside (see Supplementary Fig. S4 **c**). The strain field of T-junctions is represented as a superposition of the strain fields produced by the microtwins, as shown in Supplementary Fig. S4 **d**.

The magnetoelastic energy due to the strain field $u^{\mathrm{tw}}_{jk}(x,y)$ of the microtwins is modeled as

$$w_{\mathrm{me}} = M_s H_{\mathrm{me}}\left(u^{\mathrm{tw}}_{xx} - u^{\mathrm{tw}}_{yy}\right)(n_x^2 - n_y^2), \tag{4}$$

where the constant H_{me} characterizes the strength of the magnetoelastic coupling (expressed in the unit of the magnetic field). Here, we have omitted the terms with the strain u_{xy} since

they are irrelevant for the present model.

The equilibrium distribution of the Néel vector in the presence of the defect-induced strains minimizes the energy of the sample, given by:

$$W = \int dxdy \left\{ \frac{1}{2} A(\nabla \mathbf{n})^2 + w_{\rm an} + w_{\rm me} \right\}, \tag{5}$$

where A is the magnetic stiffness and the density of the magnetic anisotropy energy, $w_{\rm an}$, and magnetoelastic energy, $w_{\rm me}$ are defined by Eqs. (1) and (4). Orientation of the Néel vector outside nanodefects is parametrized as $n_x = \cos\theta$, $n_y = \sin\theta$. The out-of-plane component $n_z = 0$, due to strong out-of-plane anisotropy $H_{\rm an}^{\parallel} \gg H_{\rm an}^{\perp}$. We disregard any inhomogenuity the z-direction and consider θ as a function of x, y coordinates within the (001) plane.

The spatial distribution of $\theta(x, y)$ is obtained using the Euler-Lagrange equation for the energy functional Eq. (5) with the given distribution of the strain field $u_{jk}^{\rm tw}(x, y)$, which reads

$$-x_{\rm DW}^2 \Delta\theta + \frac{1}{2}\sin 4\theta + \lambda f^{\rm tw}(x, y) \sin 2\theta = 0. \tag{6}$$

In Eq. (6) we introduced the two parameters relevant for the model: domain wall width $x_{\rm DW} \equiv \sqrt{A/H_{\rm an}^{\perp}}$ and ratio of magnetoelastic energy to magnetic anisotropy $\lambda = H_{\rm me} u_0 / H_{\rm an}^{\perp}$. The dimensionless function $f^{\rm tw}(x, y)$ models distribution of the defect-induced strains.

Simulations of the domain textures given in the main text were obtained by solving equation Eq. (6) using Matlab 2021a PDE Tools with the parameters $x_{\rm DW} = 0.5$ μm and $\lambda = 10$.

References

(1) Wadley, P.; Edmonds, K. W.; Shahedkhah, M. R.; Campion, R. P.; Gallagher, B. L.; Zelezny, J.; Kunes, J.; Novak, V.; Jungwirth, T.; Saidl, V.; Nemec, P.; Maccherozzi, F.; Dhesi, S. S. Control of antiferromagnetic spin axis orientation in bilayer Fe/CuMnAs

films. *Sci. Rep.* **2017**, *7*, 11147.

(2) van der Laan, G.; Telling, N. D.; Potenza, A.; Dhesi, S. S.; Arenholz, E. Anisotropic x-ray magnetic linear dichroism and spectromicroscopy of interfacial Co/NiO(001). *Phys. Rev. B* **2011**, *83*, 064409.

(3) Pietsch, U.; Holy, V.; Baumbach, T. *High-Resolution X-Ray Scattering: From Thin Films to Lateral Nanostructures*; Advanced Texts in Physics; Springer New York, 2004.

(4) Krizek, F. et al. Molecular beam epitaxy of CuMnAs. *Phys. Rev. Materials* **2020**, *4*, 014409.

(5) Teodosiu, C. *Elastic Models of Crystal Defects*; Springer Berlin Heidelberg: Berlin, Heidelberg, 1982.
