# Supplementary material for: Defect-driven antiferromagnetic domain walls in CuMnAs films
Source: Nat Commun. 2022 Feb 7;13:724. doi: 10.1038/s41467-022-28311-x (PMC8821625; doi:10.1038/s41467-022-28311-x)
Supplement: Supplementary file 3 — Description of Additional Supplementary Files [file 41467_2022_28311_MOESM3_ESM.pdf]

Title: Supplementary Movie 1

Description: RSM of the CuMnAs 003 Bragg peak. RSMs obtained from a microtwinfree area (left) and from an area with microtwins (right) for viewing angles around the surface normal. The vertical direction is along $q_{[001]}$, The isosurface is shown in green and planar projections of the RSM are shown in the two panels.
