# Supplementary figures and images for: Defect-driven antiferromagnetic domain walls in CuMnAs films
Source: Nat Commun. 2022 Feb 7;13:724. doi: 10.1038/s41467-022-28311-x (PMC8821625; doi:10.1038/s41467-022-28311-x)

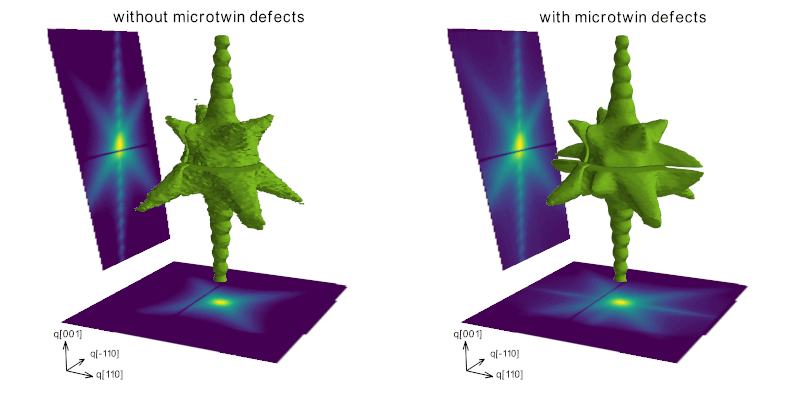

Supplement: Supplementary file 4 — Supplementary Video 1 [file 41467_2022_28311_MOESM4_ESM.gif]
